# Navigating complex cardiac complications: A case report of alcoholic cardiomyopathy and right atrial thrombosis

**DOI:** 10.1097/MD.0000000000039443

**Published:** 2024-08-23

**Authors:** Fares Abboud, Ranim Nakhal, Afif Alshwaiki, Majd Hanna, Khachig Ishkhan

**Affiliations:** a Faculty of Medicine, Damascus University, Damascus, Syrian Arab Republic; b Department of Pathology, Faculty of Medicine, Damascus University, Damascus, Syrian Arab Republic; c Stemosis for Scientific Research, Damascus, Syrian Arab Republic; d Faculty of Medicine, Aleppo University, Aleppo, Syrian Arab Republic.

**Keywords:** alcoholic cardiomyopathy, case report, right atrial thrombus, transesophageal echography, transthoracic echography

## Abstract

**Rationale:**

Alcoholic cardiomyopathy (ACM) is associated with various cardiac complications, but the development of isolated right atrial (RA) thrombus without deep vein thrombosis is rare and presents diagnostic challenges.

**Patient concerns:**

A 53-year-old Hispanic male presented with shortness of breath, chills, cough, bilateral lower extremity edema, and distended abdomen.

**Diagnoses:**

The patient was diagnosed with ACM, liver cirrhosis, and a large RA thrombus. Initial transthoracic echocardiography showed severe left ventricular systolic dysfunction but failed to detect the RA mass. Subsequent computed tomography scan and transesophageal echocardiography revealed a large oval mass in the RA, measuring 40 mm × 22 mm × 18 mm.

**Interventions:**

The patient received guideline-directed medical therapy for heart failure and anticoagulation with enoxaparin. He underwent cardiac catheterization for mechanical thrombectomy, which was minimally successful.

**Outcomes:**

The patient’s condition was managed with the prescribed interventions. Regular follow-up was planned to assess thrombolysis.

**Lessons:**

RA thrombosis is an uncommon complication of ACM. A multimodal imaging approach, with a low threshold for transesophageal echocardiography, is crucial in evaluating patients with ACM who present with cardiac complications. This approach enables accurate diagnosis and management of rare conditions like isolated RA thrombosis.

## 1. Introduction

Alcoholic cardiomyopathy (ACM) is a non-ischemic dilated cardiomyopathic disorder (DCM) and accounts for 10% of all cases of dilated cardiomyopathies.^[1]^ It is associated with many cardiac problems, including dilated cardiomyopathy, Arrhythmias, and thromboembolic events.^[2]^ The pathophysiological reasons behind these complications, precisely the development of standalone right atrial (RA) thrombus in the absence of deep vein thrombosis (DVT) remain largely unreported and presents a diagnostic and management challenge.^[3,4]^ RA thrombosis is a rare finding compared to Left atrial thrombosis. The exact prevalence is still under debate but generally varies from 0.4% to 7.5%.^[5,6]^ Transthoracic echocardiography (TTE) is a commonly used imaging tool for diagnosing Cardiac Structure and function in patients with heart failure. However, the ability of TTE to detect RA masses in the presence of alcoholic cardiomyopathy and liver cirrhosis, remains a subject of interest and clinical significance.^[7,8]^ In this presented paper, we report a case of a 53-year-old Hispanic male who was diagnosed with a very large RA thrombus predisposed by alcoholic cardiomyopathy which showed how TTE was limited in evaluating RA masses compared to transesophageal echocardiography (TEE).

## 2. Case presentation

A 53-year-old Hispanic male with no past medical history presents to the Emergency Department in a US-based hospital with a chief complaint of shortness of breath. The patient reported chills, nonproductive cough, dyspnea both at rest and exertion with 1 pillow orthopnea, abdominal distention, and urine decrease worsening over the last 3 months. The patient denied nausea, vomiting, diarrhea, or constipation. He admits to being an avid alcoholic with daily consumption without a specified quantified amount. He denies history of DVT or pulmonary embolism (PE).

On physical examination, his abdomen was distended but non-tender, and there was bilateral lower extremity edema. While his breathing sounds were normal with no wheezes or rales.

Labs ordered: Glucose, Glycohemoglobin, Comprehensive metabolic panel, Magnesium, Phosphorus, Complete blood count, Lipid panel, Troponin I, Blood cultures, Brain natriuretic peptide, Lactic acid, Lipase, and Urinalysis with urine microscopy. Blood cultures were negative. His abnormal lab values are mentioned below (Tables 1 and 2). The unmentioned values were normal.

**Table 1:** The patient’s Blood test values.

	Blood test value	Reference range
Albumin	3.1	3.4–5.4 g/dL
ALP	176	44–147 IU/L
AST	63	8–33 U/L
Calcium	8.1	8.5–10.5 mg/dL
Creatinine	1.45	0.7–1.3 mg/dL
Total protein	5.8	6–8.3 g/dL
BUN	33	6–24 mg/dL
Glucose	164	70–100 mg/dL
HgBA1C	11.8	<5.6%

**Table 2:** The patient’s urinalysis values.

	Urinalysis value	Reference range
WBCs	26	0–2 (WBCs/hpf)
RBCs	16	0–3 (RBCs/hpf)
Protein	100	0–14 (mg/dL)
Urobilinogen	2	0.1–1.8 (mg/dL)
Bacteria	+	Negative (/hpf)
Hyaline casts	+	0–2 (/lpf)
Granular casts	+	Negative (/lpf)

**Figure 1.**
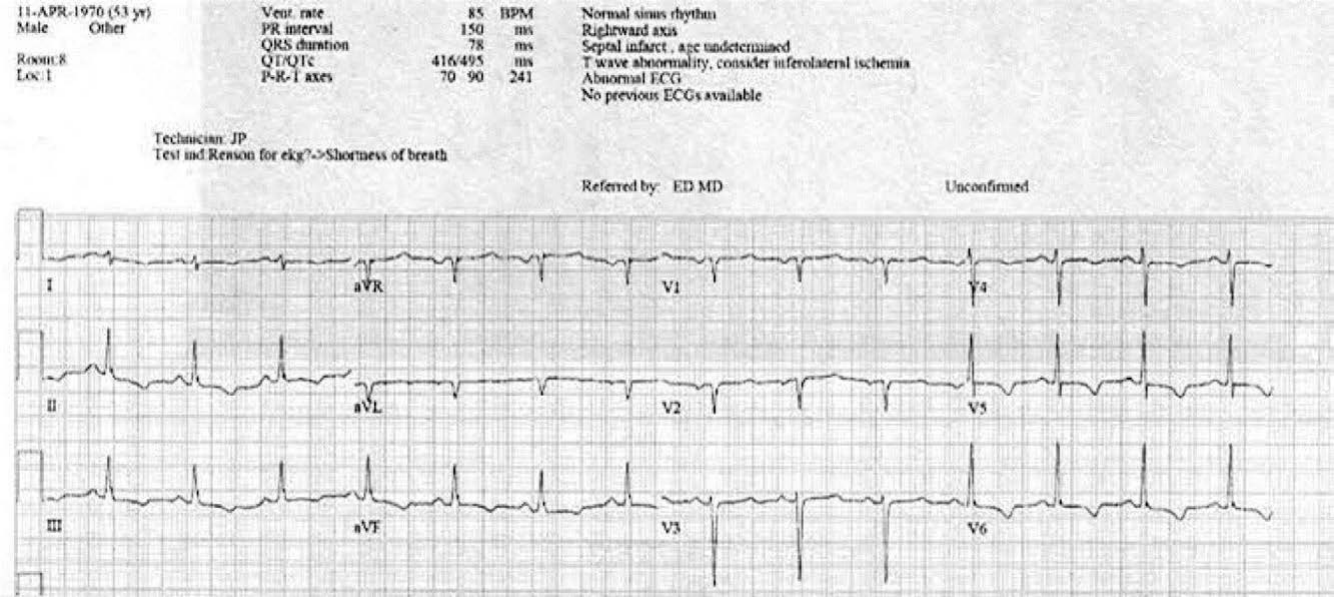
An Abnormal ECG taken on admission to the Emergency Room. ECG = electrocardiogram.

An electrocardiogram (ECG; Fig. 1) was performed which was suggestive of a septal infarct and inferior lateral ischemia with a rightward axis. A chest X-ray was unremarkable.

**Figure 2.**
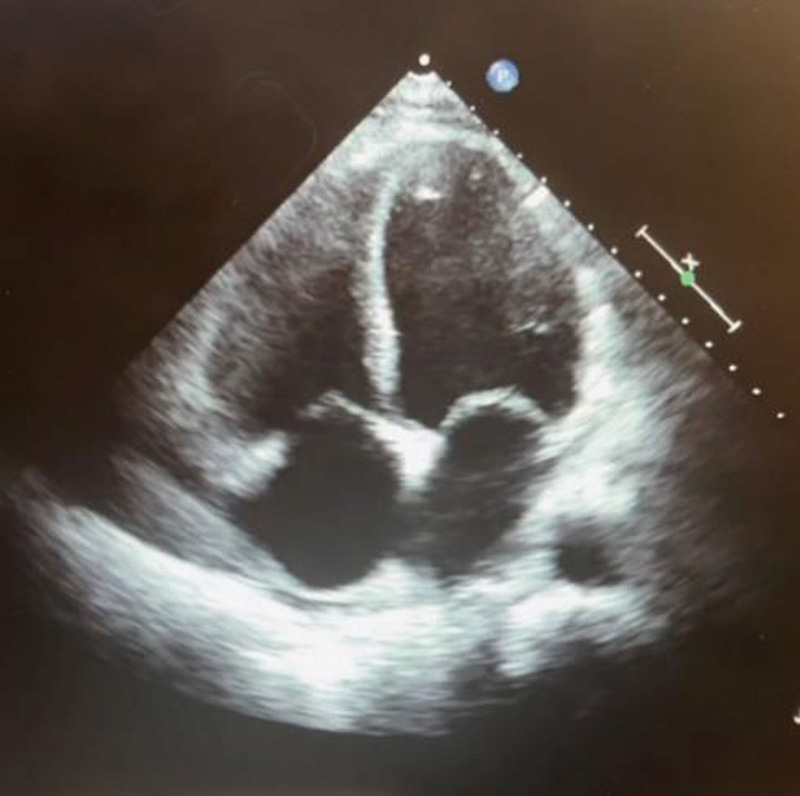
A TTE image showing the 4-chambers with no abnormalities within the RA. RA = right atrium, TTE = transthoracic echocardiography.

In addition, TTE showed a very low left ventricular ejection fraction (LVEF) of 22%, mildly dilated left ventricle (LV), and left atrium (LA) with moderate mitral regurgitation (+2), mildly dilated RA with mild tricuspid regurgitation. It showed severe LV systolic dysfunction with no evidence of any masses, acute myocardial infarction, or any abnormalities within the structure of the heart (Fig. 2). The etiology of the patient’s cardiomyopathy was most likely non-ischemic, suggestive of alcoholic cardiomyopathy.

**Figure 3.**
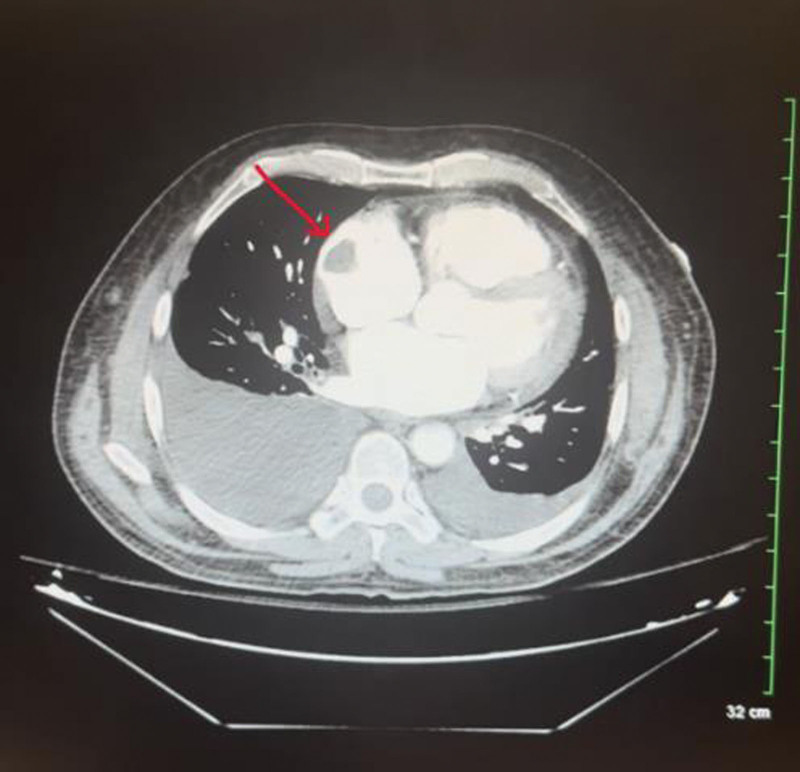
A CT scan image showing a large filling defect visible in the RA. CT = computed tomography, RA = right atrium.

Computed tomography (CT) scan of the abdomen and pelvis with Intravenous contrast showed no evidence of PE but showed lung effusions and mediastinal and hilar adenopathy. It also showed mild ascites and a large filling defect in the RA (Fig. 3).

**Figure 4.**
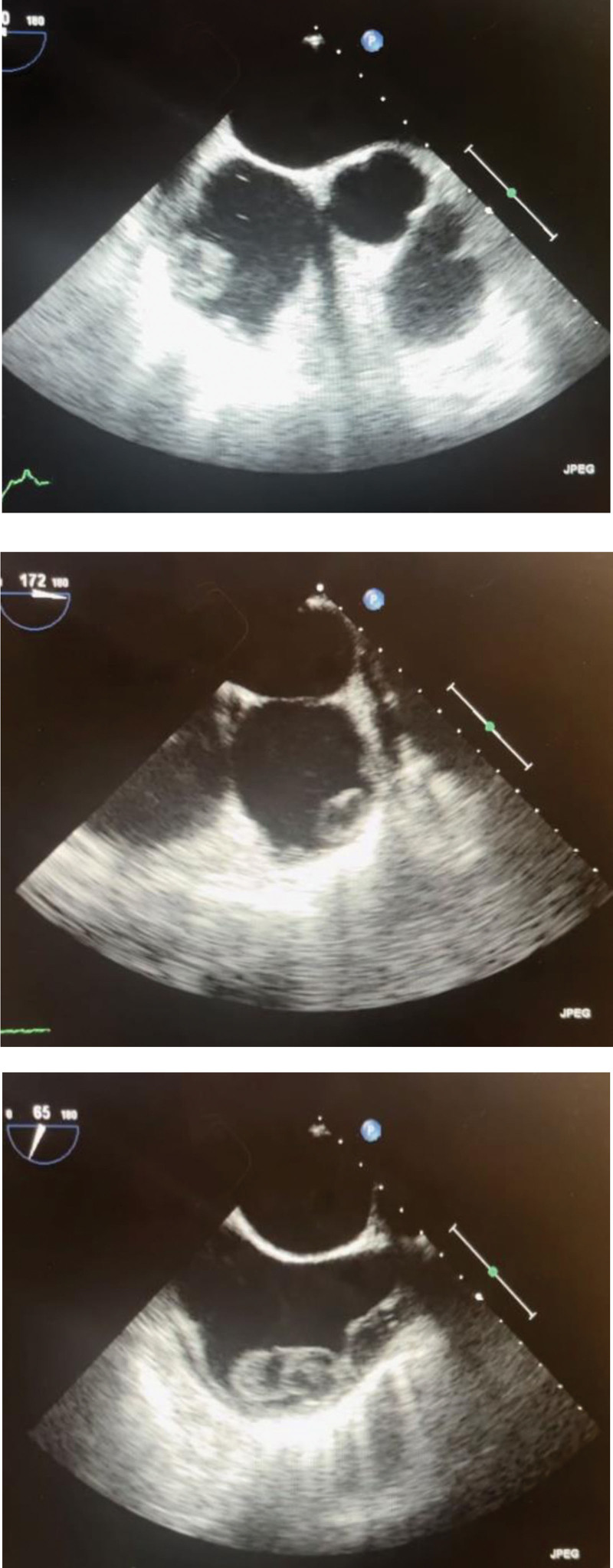
Images showing the mass in the RA from multiple views using TEE. RA = right atrium, TEE = transesophageal echocardiography.

Despite the comprehensive TTE, a large filling defect in the RA was shown on the CT scan. So, the patient went through a TEE which revealed a large oval mass measuring 40 mm × 22 mm × 18 mm attached to the lateral wall of the mildly dilated right atrium (Fig. 4). The mass did not look like endocardial vegetation. While a large thrombus was a possibility, a Tumor should be ruled out.

The patient was diagnosed with acute chronic heart failure and was administered Carvedilol tablet 6.25 mg, Lisinopril tablet 10 mg, Furosemide injection 40 mg, and Spironolactone tablet 25 mg as per Guideline therapy. His liver panel tests with the ascites were suggestive of alcoholic cirrhosis of the liver along with his alcoholic cardiomyopathy. Additionally, we administered Enoxaparin injection 40 mg due to possibility of thrombus. Patient was transferred to another facility for diagnostic cardiac magnetic resonance imaging which confirmed the RA mass as a thrombus.

The patient underwent cardiac catheterization for a mechanical thrombectomy which showed underfilled cardiac chambers at baseline with hypovolemic shock with recovery after volume resuscitation. Thrombectomy was attempted, but minimal clot was successfully aspirated. The plan is to continue anticoagulation with enoxaparin and transition to direct oral anticoagulants with regularly scheduled visits to assess thrombolysis.

## 3. Discussion

We report an unusual case of an alcoholic patient with a massive thrombus in the right atrium with mild symptoms. He was admitted to the Emergency Department with shortness of breath, chills, nonproductive cough, dyspnea, and without DVT or PE. Abnormal lab values and EKG were marked. TTE evaluation showed low LVEF, moderate mitral and tricuspid regurgitation, mildly dilated RA, and LV with systolic dysfunction without any abnormalities within the structure of the heart. A large filling defect in the RA was shown on the CT scan. However; only TEE revealed a large oval mass measuring 40 mm × 22 mm × 18 mm attached to the lateral wall of the mildly dilated RA.

Three dimensions of this rare case make it very important and need to be discussed: first, the rarity of size and thrombus location in the RA, especially with the absence of predisposing factors like DVT and PE; second, its connection with ACM and liver cirrhosis; third, the importance of TEE in diagnosing.

The RA mass incidence rate is unknown since only the symptomatic patients are referred for workup, the condition is probably underdiagnosed,^[9]^ but a review from Sweden showed that in 23,796 autopsies there was an RA thrombus rate of 7%, a rate close to the prevalence of left cardiac thrombi.^[10]^

The possible etiology of the RA masses are mostly myxoma, metastasis of extra cardiac malignant tumors such as renal cancer, intravenous leiomyomatosis, thrombosis, vegetation, and normal variants concerning the fact that they are rare findings.^[11]^ According to the evaluations of our patient, the detected mass in the RA was a thrombus following alcoholic cardiomyopathy.

ACM is a non-ischemic DCM and accounts for 10% of all cases of dilated cardiomyopathies.^[1]^

Notably, DCM is always linked with left ventricular thrombosis and systemic thromboembolic events like stroke, myocardial infarction, and peripheral arterial thromboembolism; and it is most common in the LV rather than the right heart according to its etiology.^[12,13]^ Of note, although the TTE evaluation showed low LVEF, and severe LV systolic dysfunction with no evidence of any masses, acute myocardial infarction, or any abnormalities within the structure of the heart, a large filling defect in the RA was also shown on the CT scan, no thrombi were detected in the LV. Instead, an isolated giant RA thrombus was shown by TEE in this patient.

Right heart thrombus is a rare phenomenon in the absence of structural heart disease, atrial fibrillation, catheter, or pacemaker leads located in the superior vena cava or the heart.^[14]^ The sources of the thrombi in the RA are mostly the deep veins, and it entraps in the tricuspid valve or the RV trabeculations during their intracardiac transit when the embolization occurs.^[15]^ None of the mentioned causes were detected in the history of our patient; the only possible cause was ACM in the presence of liver cirrhosis.

The major risk factor for ACM is persistent alcohol drinking, but there is no specific threshold of alcohol intake that can lead to ACM but Daily alcohol use of 80 g/d or more for more than 5 years drastically increases the risk.^[16,17]^

ACM often presents with symptoms such as worsening shortness of breath, orthopnea/paroxysmal nocturnal dyspnea, palpitations, and syncopal episodes due to tachyarrhythmias. Patients may also exhibit dilated cardiomyopathy with systolic dysfunction.^[18]^

Physical examination of patients with ACM often reveals nonspecific signs of congestive heart failure like anorexia, generalized cachexia, muscular atrophy, weakness, peripheral edema, third spacing, hepatomegaly, and jugular venous distention. Other physical signs include a displaced apical impulse and an s3 gallop sound.^[18]^

Patients with liver cirrhosis are at increased risk for bleeding and thrombosis. Because the liver synthesizes coagulation factors, anticoagulants, and the proteins involved in fibrinolysis.^[19,20]^

RA masses are commonly diagnosed with TTE. However, it is not possible to proceed with a complete examination of the whole RA with TTE, and right atrial appendage RAA is not detectable in almost all patients.^[21]^ Moreover, the limited accuracy of TTE may provide limited information about the size, composition, and mobility of the mass.^[21]^ Considering all cardiac chambers, TTE has a sensitivity of 50% to 60% for the detection of intracardiac masses,^[14]^ which decreases when considering only the RA chamber with a 60% false negative rate for the evaluation of RA thrombi and 100% misdiagnosis of RAA thrombi.^[14,21]^

TEE can reveal cardiac structures and the great vessels without any anatomic interference. In addition to the higher specificity and sensitivity in diagnosing thrombus in the heart, it also allows a direct diagnosis of PE and allows measurement of RA area and description of RAA morphology.^[14,21,22]^ Furthermore, incremental information regarding the location, size, and mobility of an RA mass, and allowing visualization from multiple angles, giving a comprehensive idea of the anatomy of the clot can be provided by three‐dimensional TEE.^[23]^ However; RAA may not be seen in 1.3% to 16% of patients.^[24,25]^

Consequently, we would recommend routinely performing a TEE examination before cardioversion with a comprehensive RA assessment, whereas in clinical practice focus is given on the LA/LAA, and RA is usually missed, and to consider TEE when the TTE window is poor and no deep vein thrombosis is detected.

Distinguishing a cardiac thrombus from a tumor-like myxoma is challenging because the clinical and radiological signs are alike, while they are the most common benign cardiac tumors 15% to 20% of which arise in the RA which is the second most commonly involved chamber after the LA cavity.^[14,26]^ CMR is currently the golden standard method for the assessment of a cardiac mass.^[27]^ And when used in this patient showed an RA thrombus with a stalk.

Regarding approach to management there are very few cases covering the best strategy in such situations. In such study measuring the long-term outcomes of treatment strategies showed that patients treated with anticoagulation had a higher risk of systemic thromboembolism (17.7%) compared to those who underwent surgical resection (0%).^[28]^

## 4. Conclusion

In summary, this case highlights the importance of a multimodal imaging approach, with a low threshold for TEE, in the evaluation of patients with alcoholic cardiomyopathy who present with cardiac complications, in order to accurately diagnose and manage rare conditions such as isolated RA thrombosis.

## Acknowledgments

We wish to show our appreciation to **Stemosis for Scientific Research**, an official Syrian Institute managed by Dr **Nafiza Martini** for the scientific environment they provide and their considerable contribution in publishing this paper.

## Author contributions

**Conceptualization:** Fares Abboud, Ranim Nakhal, Afif Alshwaiki.

**Investigation:** Fares Abboud, Ranim Nakhal, Afif Alshwaiki, Majd Hanna.

**Resources:** Khachig Ishkhan.

**Supervision:** Khachig Ishkhan

**Validation:** Khachig Ishkhan.

**Writing – original draft:** Fares Abboud, Ranim Nakhal, Afif Alshwaiki, Majd Hanna.

**Writing – review & editing:** Fares Abboud, Ranim Nakhal, Afif Alshwaiki, Majd Hanna, Khachig Ishkhan.
